# Value-Based Pricing of Health Services With Health Equity Considerations

**DOI:** 10.1016/j.jval.2025.12.014

**Published:** 2026-06

**Authors:** Luigi Siciliani, Simon Walker, David Glynn, Ni Gao, Nils Gutacker, Tim Doran

**Affiliations:** 1 Department of Economics and Related Studies, University of York, York, England, UK; 2 Center for Health Economics, University of York, York, England, UK; 3 Department of Health Sciences, University of York, York, England, UK; 4 CÚRAM Research Ireland Center for Medical Devices, Ireland; 5 Health Economics and Policy Analysis Center, University of Galway, Galway, Ireland

**Keywords:** financial incentives, health, healthcare, inequalities, pay for performance, value-based pricing

## Abstract

**Objectives:**

Many health systems aim to reduce health inequalities. Reimbursement mechanisms for healthcare providers, including pay-for-performance, are common policy levers to align provider behavior with health systems objectives. We develop a methodology to incorporate equity concerns into value-based pricing in the English National Health Service. We focus on socioeconomic inequalities in health measured by deprivation quintiles. We show how changes in the design of a national pay-for-performance scheme in primary care, the Quality Outcome Framework, can be evaluated in terms of their effects on the level and distribution of health across socioeconomic status.

**Methods:**

After developing our theoretical framework, which is based on Distributional Cost-Effectiveness Analysis and contract theory, we calibrate a model of physician behavior using information on costs and health benefits of different incentivized activities, achievement rates, and supply responsiveness to prices. We evaluate the effect of hypothetical 20% increases in price of 1 incentivized activity, which is financed by a reduction in another price to retain budget neutrality. Trade-offs between efficiency and socioeconomic inequalities in health are evaluated using the Equally Distributed Equivalent level of health.

**Results:**

We illustrate our methodology using 3 scenarios: changes in prices for 2 incentivized activities for the same health condition (diabetes), different health conditions (diabetes and chronic obstructive pulmonary disease), and across socioeconomic groups within the same activity (flu vaccination for diabetes patients).

**Conclusions:**

Our analysis illustrates how inequality aversion can be incorporated into value-based pricing when assessing the effect of financial incentives on the health distribution across socioeconomic groups.

## Highlights

• Reimbursement mechanisms for healthcare providers, including pay for performance, are common policy levers to align provider behavior with health systems objectives. We develop a methodology based on Distributional Cost-Effectiveness Analysis and contract theory to incorporate equity concerns into value-based pricing in the English National Health Service.
• Trade-offs between efficiency and socioeconomic inequalities in health after the hypothetical change of price reimbursed to providers for different activities are evaluated using the Equally Distributed Equivalent level of health.
• Our analysis illustrates how inequality aversion can be incorporated into value-based pricing when assessing the effect of financial incentives on the health distribution across socioeconomic groups.

## Introduction

Reducing health inequalities is a common policy objective.^1^^,^^2^ However, policies often focus on tackling wider social determinants of health^3^ or targeted technology adoption decisions.^4^^,^^5^ Less consideration has been given to inequalities arising from healthcare delivery and how these could be changed through redesign of delivery processes. Reimbursement mechanisms are common policy levers to direct clinical activity and align provider behavior with health systems objectives. They could be used to direct clinical time and effort toward activities that reduce health inequalities. Such an approach would need to account for any trade-offs between equity and efficiency objectives and be informed by aversion to inequality expressed by relevant stakeholders.^6^^,^^7^ Importantly, the opportunity cost of directing efforts toward one activity might involve reductions in others, which generate health benefits predominantly for certain patient population subgroups.

This article demonstrates how distributional cost-effectiveness analysis can be used to quantify these trade-offs and determine whether equity-sensitive incentives improve societal welfare.^8^ Current reimbursement mechanisms are mostly based on activity (eg, fee for service) or population served (under capitated payments). Concerns are regularly raised about under- or overprovision, and lack of quality orientation. Explicit financial incentivization in the form of pay-for-performance (P4P) schemes that relate to quality, a form of value-based pricing which aligns price with benefits,^9^ has been advocated as a solution. Such schemes redirect provider focus by linking reimbursement to measures of process quality and/or health outcomes^10^^,^^11^ with mixed success^12^, ^13^, ^14^, ^15^, ^16^, ^17^ and rarely attempt to incentivize reductions in health inequalities.^18^, ^19^, ^20^, ^21^

This study explores how equity considerations can be integrated into P4P schemes, using insights from distributional cost-effectiveness analysis^8^ and contract theory in economics.^22^, ^23^, ^24^, ^25^, ^26^ We examine how financial incentives change the health distribution across socioeconomic groups using the case study of the Quality Outcome Framework (QOF) in England, a primary wardscare P4P scheme introduced to the National Health Service (NHS) in 2004. The QOF rewards general practitioners (GPs) for meeting quality indicators, mainly for managing long-term conditions.^27^^,^^28^ Practices earn points based on the proportion of eligible patients that meet each quality target, which translates into a fee (ie, a price) for each patient. Pricing is linear, ie, the same fixed payment per patient, between a lower and upper threshold. Prices for QOF indicators are negotiated between the NHS and the British Medical Association and are not currently set with the aim of reducing health inequalities. Our analysis centers on the 2019/2020 financial year, when QOF included 68 indicators and distributed £0.7 billion in funding, averaging £102 000 per practice (8% of income).

We calibrate a model of provider behavior with evidence obtained from de novo analysis of secondary data, existing decision-analytical models, and literature. We simulate the effect of different interventions to provider reimbursement: changes in QOF bonus payments (prices) for 2 incentivized activities for the same health condition, different health conditions, and across socioeconomic groups within the same activity. The simulations illustrate how inequality aversion can be systematically considered when assessing the effect of financial incentives on the health distribution.

## Methods

### Model of Provider Behavior

We model GP provider behavior and how they respond to prices. We then compute the effect on total health and socioeconomic inequalities in health. Define $n_{jic}$ as the number of patients in GP practice *j* who receive QOF process *i* for health condition *c*; $N_{jic}$ as the number of eligible patients who could receive QOF process *i* for condition *c*; and $p_{jic}$ as the price for treating 1 more patient. Patients differ in socio-economic status (SES) $s\in \left\{s_{1},\ldots ,s_{S}\right\}$. The number of patients who receive the QOF for a given SES *s* is $n_{jics}$ with $n_{jic}=\sum_{s}n_{jics}$. Similarly, the number of eligible patients is $N_{jics}$ with $N_{jic}=\sum_{s}N_{jics}$.

GPs have altruistic concerns toward patients’ health. The payoff function of GP practice *j*, $V_{jcs}\left(.\right)$, is the sum of utility from improving patient health and financial surplus:

$V_{jcs}\left(.\right)$ = *α health + revenues − costs*, where *α* is altruism and denotes the weight given to health relative to financial surplus. The cost of attracting and providing process *i* differs across patients within condition *c*, practice *j* and SES *s*. For some patients, a simple invitation will convince them to visit the GP, whereas for others follow-up calls are required. We assume that the cost sustained by a GP practice to provide a given process, defined with *k*, varies across patients with density function $f_{jics}\left(k\right)$ over the support $k\in \left[\underline{k},\bar{k}\right]$ and cumulative density function $F_{jics}\left(k\right)$.

For a given condition *c* and SES *s* patients are homogenous in health. Patient health in GP practice *j* is $H_{jcs}\left(i=1\right)$ when the QOF process is provided, and $H_{jcs}\left(i=0\right)$ when not. We assume the QOF process (weakly) increases patient health, $H_{jcs}\left(i=1\right)\geq H_{jcs}\left(i=0\right)$. We assume that more deprived patients have lower health, $H_{jcs}\left(i=0\right)<H_{jc\left(s+1\right)}\left(i=0\right)$. In Appendix 1 in Supplemental Materials, we show that the total number of patients who receive the QOF for a given SES *s* is

$$n_{jics}^{*}=N_{jics}\;F_{jics}\left(k_{jics}^{*}\right)\text{,}$$

and varies with the number of eligible patients, the ability to benefit and the distribution of costs. The effect of increasing the QOF price $p_{jic}$ for patients with a given SES is:

$$\frac{\partial n_{jics}^{*}}{\partial p_{jic}}=N_{jics}\;f_{jics}\left(k_{jics}^{*}\right)\text{.}$$

For a given GP practice and condition, total health benefit across socioeconomic groups is

$$\sum_{s}\left\{H_{jcs}\left(i=1\right)\;n_{jics}^{*}+H_{jcs}\left(i=0\right)\;\left[N_{jics}-n_{jics}^{*}\right]\right\}\text{.}$$

Define $h_{s}^{t}$ as individual health and $Z_{s}^{t}$ as the number of individuals in the population with SES *s* at time t ={0,1}. Total health benefit at time *t* for patients with SES *s* is

$${TH}_{s}^{t}=h_{s}^{t}\;Z_{s}^{t}+\sum_{c}\sum_{i}\sum_{j}\left(H_{jics}^{t}\left(i=1\right)-H_{jics}^{t}\left(i=0\right)\right){\;n}_{jics}^{t*}\text{,}$$

which is the sum of baseline health and the additional health benefit from the QOF. Total health benefit is ${TH}^{t}=\sum_{s}{TH}_{s}^{t}$. To consider the consequences on socioeconomic inequalities in health, we use the Atkinson (social welfare) index and compute the Equally Distributed Equivalent (EDE) level of health^29^:

$${EDE}^{t}={\left[\frac{\sum_{s}\;\left[{\left({AH}_{s}^{t}\right)}^{1-\varepsilon }\;Z_{s}^{t}\;\right]}{\sum_{s}Z_{s}^{t}}\right]\;}^{\frac{1}{1-\varepsilon }}$$

in which ε is an inequality aversion index and ${AH}_{s}^{t}={TH}_{s}^{t}\;/\;Z_{s}^{t}$ is the average health. For ε=0 the index gives average health. If the policy increases average health and health inequalities, the EDE index can assess trade-offs between equity and efficiency. The effect of the intervention is ${EDE}^{1}-{EDE}^{0}\text{.}$

### Simulations

#### Change in prices within the same health condition

Define A and B the 2 affected QOF processes. Price A increases, whereas price B reduces within the same condition *c*, $p_{jAc}$ and $p_{jBc}$. Time 0 is the prepolicy period and time 1 the postpolicy period. The policy is budget neutral:

$$\sum_{j}\sum_{s}\left({p_{jAc}^{0}n}_{jAcs}^{0*}+{p_{jBc}^{0}n}_{jBcs}^{0*}\right)=\sum_{j}\sum_{s}\left({p_{jAc}^{1}n}_{jAcs}^{1*}+{p_{jBc}^{1}n}_{jBcs}^{1*}\right)\text{.}$$

A higher price increases reimbursement directly and indirectly through higher volume. Given that reimbursement is monotonic in price, for a given increase in price A it is always possible to reduce price B to make the intervention budget neutral.

In addition to affecting health, the policy could also increase NHS treatment costs over patient lifetime or reduce them if it prevents adverse events and hospitalizations. Given the NHS works within a fixed budget, if the policy intervention increases costs, this will be offset by a reduction in health spending and therefore population health elsewhere in the health system. The additional cost (saving) of the policy is

$$\Delta C=\sum_{j}\sum_{s}\left({C_{Ac}^{1}n}_{jAcs}^{1*}+{C_{Bc}^{1}n}_{jBcs}^{1*}\right)-\sum_{j}\sum_{s}\left({C_{Ac}^{0}n}_{jAcs}^{0*}+{C_{Bc}^{0}n}_{jBcs}^{0*}\right)\text{,}$$

in which $C_{ic}^{t}$ is the cost for the health system for a given health condition and process.

We assume that $\delta_{s}^{t}$ are weights at which health is reallocated by SES such that $\sum_{s}\delta_{s}^{t}=1$. The health loss for each individual in group *s* is equal to $\frac{\Delta C}{v}\delta_{s}^{t}\frac{Z_{s}^{t}}{\sum_{s}Z_{s}^{t}}$ in which v is the cost-effectiveness threshold used to convert costs into health losses.

The total health benefit at t=1 for patients with SES *s* is

$${TH}_{s}^{1}=\left(h_{s}^{1}-\frac{\Delta C}{v}\delta_{s}^{t}\frac{Z_{s}^{1}}{\sum_{s}Z_{s}^{1}}\right)\;Z_{s}^{1}+\sum_{c}\sum_{i}\sum_{j}\left(H_{jics}^{1}\left(i=1\right)-H_{jics}^{1}\left(i=0\right)\right){\;n}_{jics}^{1*}\text{.}$$

#### Change in prices across health conditions

The same methodology can be applied to increase the price within a health condition financed by a price reduction from a different health condition, in which $p_{jA\left(c1\right)}$ and $p_{jB\left(c2\right)}$ are the prices for process A and B within conditions 1 and 2.

#### Change in prices across socioeconomic groups

The price can also vary by SES $p_{jics}$. With five deprivation quintiles, the price vector before and after policy is $p_{jics}^{0}=\left\{p_{jic1}^{0},\ldots ,p_{jic5}^{0}\;\right\}$ at t=0 and as $p_{jics}^{1}=\left\{p_{jic1}^{1},\ldots ,p_{jic5}^{1}\;\right\}$ at t=1.

### Parameterization

We conduct 3 simulations to illustrate ways of incentivizing equity improvements within the QOF scheme. In each simulation, we ensure changes are QOF budget neutral.

#### Patient populations and QOF indicators

We focus on patients with diabetes (types 1 and 2) and chronic obstructive pulmonary disease (COPD) and consider 3 QOF indicators for 2019/2020: influenza vaccination for patients with diabetes (DM018), influenza vaccination for COPD patients (COPD007), and structured education program for diabetes patients (DM014). The influenza indicators are met if an eligible patient was vaccinated against influenza between 1 August 2019 and 31 March 2020. The education indicator is met if a patient newly diagnosed with diabetes (between 1 April 2019 and 31 March 2020) is referred to a structured education program within 9 months of entering the practice diabetes register, regardless of take-up. The number of eligible patients for each QOF indicator and for whom it was achieved were obtained from 300 randomly selected primary-care practices in the clinical practice research Datalink (CPRD) Aurum database^30^ (Appendix 2 in Supplemental Materials).

#### Socioeconomic deprivation

Patients were assigned to deprivation quintile groups based on the Index of Multiple Deprivation (IMD) score for their area of residence^31^ (Appendix 2 in Supplemental Materials).

#### Health benefits and costs

For each QOF indicator, we used previously developed decision-analytical models to estimate health and cost impacts of a patient receiving the incentivized process conditional on their IMD score by accounting for socioeconomic differences in individual characteristics and model parameters. Evidence on effectiveness and cost of indicator achievement were sourced from National Institute for Health and Care Excellence (NICE) clinical guidelines or targeted literature reviews. Health was captured using quality-adjusted life-years (QALYs). Costs were estimated from the perspective of the NHS and personal social services, separated into QOF delivery and wider NHS costs (eg, resulting from hospitalizations). QALYs and costs were discounted at an annual rate of 3.5%.^32^

Costs and health benefits for individuals with diabetes were calculated using a cost-effectiveness model based on the UK Prospective Diabetes Study Outcomes Model 2. The model captures the risk of death and 8 diabetes-related clinical events and associated morbidity and mortality.^33^ Differences in risk of baseline events by deprivation group were estimated from the National Diabetes Audit (2010-2011).^34^ Quality of life data were from Alva et al.^35^ Baseline patient covariates for the risk model were based on primary care data from The Health Improvement Network. To capture the impact of influenza vaccination on QALYs, an estimate was based on reduced probability of influenza infection and reductions in morbidity and mortality. To capture the impact of a structured education program, the IMD-adjusted probability of taking up a program if referred was combined with efficacy of structured education in terms of reduced blood glucose, blood pressure, and body mass index.^36^ Health care costs, including diabetes complications, were from Alva et al^35^ updated to 2019/2020 using the NHS cost inflation index.^37^ To adjust costs for IMD we used multipliers from Asaria,^38^ which provides IMD estimates for costs conditional on age and sex.

COPD costs and health benefits were estimated based on cost-effectiveness models developed for the NICE COPD clinical guideline. The model captures COPD disease progression based on worsening lung function, classified by Global Initiative for Chronic Obstructive Lung Disease (GOLD) stage measured using forced expiratory volume. We captured the distribution of patients in each GOLD state by IMD based on adjusting the mean forced expiratory volume observed in the UK COPD population^39^ by an IMD multiplier derived from McAllister et al^40^ to estimate the mean by IMD and then applied a distribution to these based on observed population average. The model applies a constant health-related quality of life score for each GOLD stage. We adjusted these scores by IMD using multipliers derived from Schneider et al^41^ to reflect deprivation differences. The model also captured health-related quality of life reductions from exacerbations and adverse events. Five cost categories were included: drug costs, maintenance costs, exacerbation costs, adverse event costs, and treatment progression. These varied with severity of COPD and are expressed in 2019/2020 GBP. As with diabetes, the impact of influenza vaccination was based on the reduced probability of influenza infection and reductions in morbidity and mortality.

Empirical estimates suggest that in England one QALY is displaced for every £15 000 increase in NHS expenditure.^42^

These “health opportunity costs” are not equally distributed in the population with those in more deprived areas disproportionately bearing these costs.^43^ We use these weights to distribute opportunity costs by IMD.

Table 1 describes QALYs, future NHS costs and number of patients by QOF indicator and achievement status. Table 2 provides a summary of the main variables, model parameters, and corresponding sources.

**Table 1:** Health benefit, future NHS costs, and indicator achievement for selected QOF indicators.

Deprivation quintile group	Diabetes						Chronic obstructive pulmonary disease (COPD)		
	Influenza vaccination (DM018)			Education program (DM014)			Influenza vaccination (COPD007)		
	Indicator met	Indicator not met	Delta	Indicator met	Indicator not met	Delta	Indicator met	Indicator not met	Delta
QALYs (discounted)									
IMD1-most deprived	7.263	7.237	0.026	8.802	8.795	0.007	4.229	4.219	0.010
IMD2	8.058	8.027	0.031	9.826	9.816	0.010	4.875	4.863	0.013
IMD3	9.223	9.184	0.039	11.191	11.182	0.009	5.478	5.463	0.016
IMD4	9.551	9.510	0.041	11.585	11.575	0.010	5.794	5.776	0.017
IMD5-least deprived	10.184	10.137	0.047	12.291	12.280	0.011	6.187	6.168	0.019
Future NHS costs (discounted; in 2020 GBP)									
IMD1-most deprived	43 558	43 414	145	47 777	47 712	65	28 002	27 913	89
IMD2	39 861	39 709	151	42 868	42 796	72	29 972	29 873	99
IMD3	37 673	37 513	160	39 578	39 509	69	30 574	30 469	105
IMD4	35 854	35 691	163	37 698	37 642	56	31 111	31 002	109
IMD5-least deprived	35 098	34 929	169	36 554	36 493	61	31 918	31 804	114
Indicator achievement (300 practices; Financial year 2019/20)									
IMD1-most deprived	22 688	2580	89.8%	1576	172	90.2%	10330	805	92.8%
IMD2	23 528	2536	90.3%	1431	163	89.8%	9074	593	93.9%
IMD3	20 691	1784	92.1%	1247	152	89.1%	7299	411	94.7%
IMD4	19 757	1531	92.8%	1112	120	90.3%	6800	283	96.0%
IMD5-least deprived	17 408	1296	93.1%	955	112	89.5%	5507	229	96.0%

Delta indicates difference between “indicator met” and “indicator not met”; IMD, index of multiple deprivation; QALY, quality-adjusted life-years.

**Table 2:** Source of model parameters.

Variable	Model parameter	Source
Health benefits and costs, diabetes, influenza vaccination	H,C	Decision-analytical models^33^^,^^35^
Health benefits and costs, diabetes, structured education program	H,C	Decision-analytical models; Deakin et al. (2014)
Health benefits and costs, COPD	H,C	Cost-effectiveness models; NICE (2019), McAllister et al.^40^
Number of eligible patients	N	CPRD Aurum database^30^
Number of eligible patients for whom the QOF indicator is achieved	n	CPRD Aurum database^30^
Socioeconomic deprivation	s	Index of Multiple Deprivation^31^
Provider reimbursement	p	Authors’ calculations based on 2019/20 GMS contract (2019)
Supply elasticity	$\frac{\partial n}{\partial p}\;\frac{p}{n}$	Regression-based estimates^44^
Inequality aversion	ε	Experiment design^7^
Cost-effectiveness threshold	v	Lomas et al.^42^
Weights of health opportunity costs by deprivation	δ	Love-Koh et al.^43^

#### Provider reimbursement

GP practice reimbursement per patient who received influenza vaccination was £3.55 in the diabetes population (DM018) and £20.11 in the COPD population (COPD007). The reimbursement for referral to a structured education program was £181.86 (DM014).

#### Elasticity of GP supply

The degree to which GPs respond to price by altering volume is the supply elasticity, which can differ across deprivation and QOF indicators. Based on, Lomas et al^44^ we used an elasticity of 0.17 for the 2 most-deprived quintiles and of 0.15 for the 3 least-deprived quintiles (see Appendix 3 in Supplemental Materials for details).

#### Inequality aversion

We assumed inequality aversion in the general population of e = 3.5 based on the latest evidence for England^7^ and conduct threshold analysis over a range of values.

#### Sensitivity analyses

We conducted 1-way sensitivity analyses to explore uncertainty in key modeling assumptions and their impact on the simulated change in EDE after incentive redesign. We model individual scenarios in which we either (1) vary the elasticity of GP supply (by ±20% on the base values), (2) vary the marginal QALY gain from receiving incentivized QOF care (by ±20%), (3) assume no socioeconomic gradient in the QALY gain from incentivized QOF care (ie, all IMD groups are assumed to gain as much as the least-deprived IMD quintile), (4) assume higher opportunity cost of NHS expenditure of £30 000 per QALY in line with current NICE guidelines for technology assessment, or (5) assume an equal distribution of opportunity costs across all IMD groups in the population.

## Results

### Simulation 1. Price changes across diabetes indicators

We investigate the effect of a 20% increase in price reimbursed to GPs for vaccinating patients with diabetes against influenza (QOF indicator DM018; from £3.55 to £4.27). This is financed by a price reduction for patients with diabetes who were referred to structured education programs (DM014) of 6.7% (from £181.66 to £169.67).

**Figure 1:**
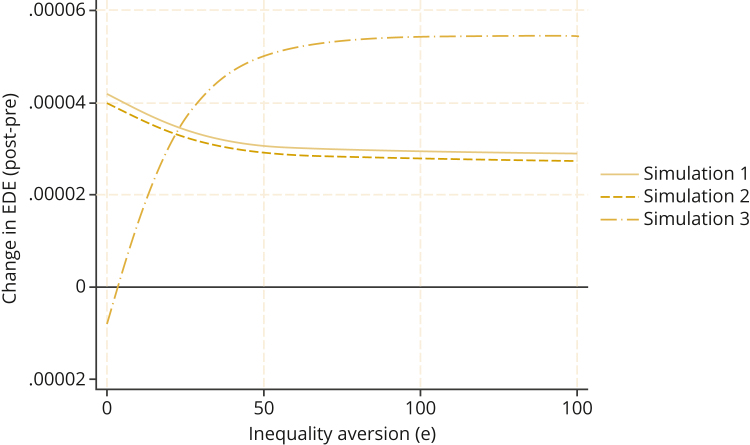
Change in Equally Distributed Equivalent (EDE) level of health in the English population after incentive change, calculated at different levels of inequality aversion.

Because an increase in the uptake of vaccination generates more QALYs than a referral to structured education programs, the change in reimbursement increases population health by 2630 QALYs and increases long-term NHS expenditure by 11.5m GBP, equivalent to a population health loss of 765 QALYs (assuming a marginal productivity of NHS expenditure of 15 000 GBP per QALY) (Table 3). The net increase in population health is 1865 QALYs. The effect is positive in each IMD group but larger for the least-deprived quintile. Hence, the simulated adjustment increases both overall population health and health inequalities. The gains in efficiency outweigh the losses in equity, thus increasing social welfare. The change in EDE is positive even for implausibly high levels of inequality-aversion parameters, eg, above 50 (Fig. 1).

**Table 3:** Effect of incentive adjustment on health in targeted patient population and general population in England: Simulations.

IMD group	Effect of QOF change (in QALYs)			Average health (per person) (in QALYs)	
	Health effect	Expenditure effect	Change in total health (population)	Baseline	Change
Simulation 1					
Most deprived (IMD1)	444	−202	242	62.36	0.000029
IMD2	552	−167	384	65.52	0.000042
IMD3	540	−167	373	69.49	0.000041
IMD4	548	−122	426	70.64	0.000047
Least deprived (IMD5)	546	−106	440	73.02	0.000050
Total	2630	−765	1865	68.25	0.000042
Simulation 2					
Most deprived (IMD1)	408	−179	229	62.36	0.000027
IMD2	512	−149	363	65.52	0.000040
IMD3	505	−148	357	69.49	0.000039
IMD4	512	−109	403	70.64	0.000045
Least deprived (IMD5)	513	−94	419	73.02	0.000048
Total	2450	−679	1771	68.25	0.000040
Simulation 3					
Most deprived (IMD1)	447	5	452	62.36	0.000054
IMD2	0	4	4	65.52	0.000000
IMD3	0	4	4	69.49	0.000000
IMD4	0	3	3	70.64	0.000000
Least deprived (IMD5)	−776	3	−773	73.02	−0.000089
Total	−329	20	−309	68.25	−0.000007

IMD indicates index of multiple deprivation; QALY, quality-adjusted life-years; QOF, quality outcome framework.

### Simulation 2. Price changes across diseases

Consider a 20% price increase for influenza vaccination in diabetes patients (DM018) financed by a price reduction for the same activity for COPD patients (COPD007) by 9.8% (£1.97). The simulated adjustment again increases both overall population by 1771 QALYs and health inequalities (Table 3). The change in EDE is positive even for very high levels of inequality-aversion parameters (Fig. 1).

### Simulation 3. Price changes across deprivation groups

Last, we simulate the effect of increasing the price for influenza vaccination in diabetes patients (DM018) by 20% for the most-deprived quintile, who on average gain least from vaccination, which is financed by a 28.3% reduction in price for the same indicator for the least-deprived quintile, who on average gain most from vaccination.

The intervention reduces health among diabetes patients by 329 QALYs (Table 3). There are no direct health effects on patients in IMD quintile groups 2 to 4, for which the price remains unchanged. Long-term NHS expenditure reduces overall, which results in 20 additional QALYs generated elsewhere in the system that fall mostly on the most deprived.

The intervention reduces overall population health but results in a more even distribution of average health across IMD groups. Figure 1 suggests that the simulated intervention increases social welfare for all inequality aversion parameters e ≥ 3.3, which is consistent with current estimates of inequality aversion in England.^7^

### Sensitivity analyses

**Figure 2:**
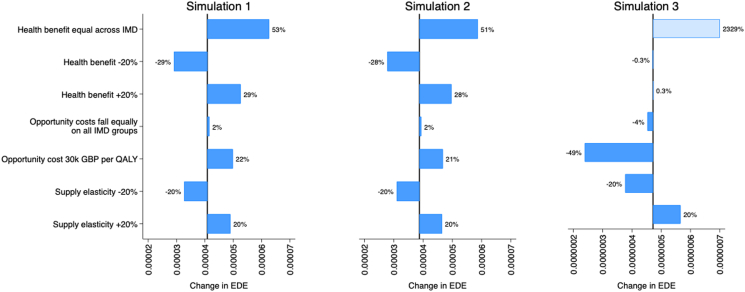
Sensitivity analyses. The solid black line denotes the change in EDE after the incentive redesign in our main (base-case) scenario reported in Table 3. Each bar denotes a sensitivity analysis, and the length of the bar denotes the absolute change from base case. The number next to each bar denotes the relative change from base case in percent. The light bar in the scenario analysis for simulation 3 denotes a larger change from base case than could be plotted here (Δ = 0.0000115), and the bar is capped at the end of the scale.

Figure 2 presents tornado diagrams of the sensitivity analyses to each simulation (Appendix 4 in Supplemental Materials). All 3 simulations are most sensitive to the assumption of differential QALY gain arising from receipt of QOF care, ie, patients’ ability to generate health from healthcare. However, the estimated change in EDE remains positive in all scenarios and for all incentive changes explored.

## Discussion

We have developed a method to account for health inequalities by socioeconomic status when evaluating aspects of financial incentive designs aimed at improving healthcare provider performance. Our analysis demonstrates that health inequality aversion can be incorporated into value-based pricing when assessing the effect on the health distribution across socioeconomic groups. However, the approach is informationally demanding because it requires knowledge of key parameters such as health gains of interventions, supply elasticity to prices by SES, and estimates of health inequality aversion.

Previous evaluations of the QOF have generally found modest improvements in quality of care but limited evidence of reductions in socioeconomic inequalities in health outcomes.^14^ Our findings suggest that uniform incentive structures may not be sufficient to address existing health inequalities and that differential pricing targeted at activities or populations with greater potential to reduce inequalities may yield more equitable outcomes within existing budgets (a national policy priority in England^45^^,^^46^), although this may come at the cost of overall efficiency. Our approach makes these trade-offs explicit and shows how varying degrees of inequality aversion affect the ranking of alternative policy options, allowing for informed decision making.

The study has some limitations. First, we only investigated a limited number of trade-offs. Many more could be conducted with the relevant information. Second, our analysis is positive rather than normative; we did not derive the “optimal” set of prices to improve population EDE but instead considered potential changes to an existing incentive scheme. Third, we did not formally allow for multimorbidity, which is low in our sample (5.3% of patients have diabetes and COPD) or double payments across conditions. Fourth, we only explored uncertainty in a subset of modeling assumptions. Fifth, our analysis relies on estimates of health inequality aversion but methods for eliciting public levels of aversion are still evolving.^47^

Future research could extend this work in several ways. First, assumptions underlying our analysis could be refined through further qualitative and quantitative research on health inequality aversion in the population, with the aim of reaching consensus on the appropriate degree of reweighting of benefits. Second, the approach could be broadened to include a wider range of clinical activities and conditions, as well as other dimensions of inequality, such as ethnicity, geography, and socioeconomic status. This would require enhanced data collection and linkage of the relevant variables at the patient level, which would need to be standardized, complete, accurate, timely, and frequently updated.^48^ This would also allow policymakers and researchers to better track progress toward reducing health inequalities, identifying emerging inequalities and adjusting interventions as necessary. Third, more detailed modeling of provider behavior could account for heterogeneity across practices and local health economies, informed by estimates of supply elasticities by socioeconomic status. Fourth, future studies could explore the integration of this framework into routine NHS pricing and commissioning processes, including its interaction with other policy levers such as capitation and block contracts. Fifth, evaluations of actual policy changes designed using this approach could assess its practical effectiveness and feasibility, including acceptability to incentivized physicians and their patients. Sixth, alternative methods such as probabilistic sensitivity analysis^49^ to capture parameter uncertainty. Last, current financial incentives target healthcare processes and quality of care, but they do not vary by SES. Future work could further investigate these issues.

## Conclusion

The effects of financial incentives on the health distribution across socioeconomic groups can be assessed by incorporating inequality aversion into value-based pricing frameworks.

## Article and Author Information

**Authorship Confirmation:** All authors certify that they meet the ICMJE criteria for authorship.

**Funding/Support:** This research was funded by the National Institute for Health and Care Research (NIHR) Policy Research Program (project reference NIHR201728). The views expressed are those of the author(s) and not necessarily those of the NIHR or the Department of Health and Social Care. David Glynn received the financial support of the European Union’s Horizon Europe Excellent Science program under the Marie Skłodowska-Curie Actions Grant Agreement (Grant Agreement No 101081457) and in part from Research Ireland under grant number 13/RC/2073_P2.

**Role of the Funder/Sponsor:** The funder had no role in the design and conduct of the study; collection, management, analysis, and interpretation of the data; preparation, review, or approval of the manuscript; or decision to submit the manuscript for publication.

## Author Disclosures

Author disclosure forms can be accessed below in the Supplemental Material section.

## Supplemental Material

Author Disclosure
