# Supplementary material for: Value-Based Pricing of Health Services With Health Equity Considerations
Source: Value Health. 2026 Jun;29(6):1004–11. doi: 10.1016/j.jval.2025.12.014 (PMC13259816; doi:10.1016/j.jval.2025.12.014)
Supplement: Author Disclosure [file mmc1.pdf]

Tim Doran

Discloser Identifier: 55022126
Disclosure Purpose: VIH-2025-0718.R1 - Value-based Pricing of Health Services with Health Equity Considerations.

Employment Information: Currently Employed

Summary of Interests

Company or Organization

Entity	Type	Relevant to this Disclosure
**National Institute for Health Research**	Grant / Contract	Yes
**University of York**	Employment Current Employment	
**Title:** Professor of Health Policy		

Additional Questions

1. **Please select which of the following apply to each relationship or activity:**
   a. **Employment** University of York

      The relationship is in direct support of the work reported in the manuscript anytime from when the work was conceived

   b. **Grant / Contract** National Institute for Health Research

      The relationship is in direct support of the work reported in the manuscript anytime from when the work was conceived

2. **I confirm I have disclosed all direct support for the present manuscript (e.g. funding, provision of study materials, medical writing, article processing charges, etc.) There is no time limit for this item.**

   Yes

3. **Please indicate below whether in the past 36 months you have had any of the following interests that are topically related to the work reported in the manuscript.**
   a. **Employment (If you need to add an interest, please scroll to the top of the page, click "add interest" and select “Employment”)**

      No, I have no relevant interests of this type

   b. **Grants or contracts for research (If you need to add an interest, please scroll to the top of the page, click "add interest" and select “Grant/Contract”)**

      No, I have no relevant interests of this type

   c. **Payment for consulting (If you need to add an interest, please scroll to the top of the page, click "add interest" and select “Independent Contractor”)**

      No, I have no relevant interests of this type

   d. **Payments or honoraria for lectures, presentations, speakers bureaus, or educational events (If you need to add an interest, please scroll to the top of the page, click "add interest" and select "Independent Contractor" and include the correct information under "Consultant")**

      No, I have no relevant interests of this type

   e. **Payment for service on an advisory board (If you need to add an interest, please scroll to the top of the page, click "add interest" and select “Independent Contractor,” and choose “Other”)**

      No, I have no relevant interests of this type

   f. **Payment for participation Data and safety monitoring board (If you need to add an interest, please scroll to the top of the page, click "add interest" and select “Independent Contractor”)**

      No, I have no relevant interests of this type

g. **Expert witness testimony (If you need to add an interest, please scroll to the top of the page, click "add interest" and select "Independent Contractor")**

No, I have no relevant interests of this type

h. **Royalties from Patents, Trademarks, Copyrights or other intellectual property (If you need to add an interest, please scroll to the top of the page, click "add interest" and select the appropriate interest type)**

No, I have no relevant interests of this type

i. **Patents planned, issued, or pending, whether or not you receive royalties (If you need to add an interest, please scroll to the top of the page, click "add interest" and select "Patents")**

No, I have no relevant interests of this type

j. **Fiduciary Officer or Other Board Membership (If you need to add an interest, please scroll to the top of the page, click "add interest" and select "Fiduciary Officer")**

No, I have no relevant interests of this type

k. **Stock or stock options (If you need to add an interest, please scroll to the top of the page, click "add interest" and select the appropriate interest type)**

No, I have no relevant interests of this type

l. **Support for attending meetings or other travel (If you need to add an interest, please scroll to the top of the page, click "add interest" and select "Travel")**

No, I have no relevant interests of this type

4. **Was any individual paid to provide professional writing assistance with this manuscript?**

No.

5. **Have you or your institution received equipment, materials, drugs, or services in direct support of the work in the manuscript (without time limit) not disclosed above?**

No.

6. **In the past 36 months, have you received equipment, materials, drugs, medical writing, gifts or other services from for-profit or not-for-profit third parties whose interests may be affected by the content of the manuscript not disclosed above?**

No.

7. **Are there other financial or non-financial interests that readers could perceive to have influenced, or that give the appearance of potentially influencing, what you wrote in the submitted work not disclosed above.**

No.

Certification

I certify that I have answered every question and the information provided in this disclosure is complete and accurate.

Convey
Global Disclosure System
AAMC

Ni Gao

Discloser Identifier: 105749454
Disclosure Purpose: VIH-2025-0718.R1 - Value-based Pricing of Health Services with Health Equity Considerations.

Employment Information: Currently Employed

Summary of Interests

Company or Organization

Entity	Type		Relevant to this Disclosure
**University of York**	Employment	Current Employment	Yes

Title: Research Fellow

Additional Questions

1. **Please select which of the following apply to each relationship or activity:**
   a. **Employment** University of York

      The relationship is in direct support of the work reported in the manuscript anytime from when the work was conceived
2. **I confirm I have disclosed all direct support for the present manuscript (e.g. funding, provision of study materials, medical writing, article processing charges, etc.) There is no time limit for this item.**

   Yes
3. **Please indicate below whether in the past 36 months you have had any of the following interests that are topically related to the work reported in the manuscript.**
   a. **Employment (If you need to add an interest, please scroll to the top of the page, click "add interest" and select "Employment")**

      Yes, as disclosed above

   b. **Grants or contracts for research (If you need to add an interest, please scroll to the top of the page, click "add interest" and select "Grant/Contract")**

      No, I have no relevant interests of this type

   c. **Payment for consulting (If you need to add an interest, please scroll to the top of the page, click "add interest" and select "Independent Contractor")**

      No, I have no relevant interests of this type

   d. **Payments or honoraria for lectures, presentations, speakers bureaus, or educational events (If you need to add an interest, please scroll to the top of the page, click "add interest" and select "Independent Contractor" and include the correct information under "Consultant")**

      No, I have no relevant interests of this type

   e. **Payment for service on an advisory board (If you need to add an interest, please scroll to the top of the page, click "add interest" and select "Independent Contractor," and choose "Other")**

      No, I have no relevant interests of this type

   f. **Payment for participation Data and safety monitoring board (If you need to add an interest, please scroll to the top of the page, click "add interest" and select "Independent Contractor")**

      No, I have no relevant interests of this type

   g. **Expert witness testimony (If you need to add an interest, please scroll to the top of the page, click "add interest" and select "Independent Contractor")**

      No, I have no relevant interests of this type

h. **Royalties from Patents, Trademarks, Copyrights or other intellectual property (If you need to add an interest, please scroll to the top of the page, click "add interest" and select the appropriate interest type)**

No, I have no relevant interests of this type

i. **Patents planned, issued, or pending, whether or not you receive royalties (If you need to add an interest, please scroll to the top of the page, click "add interest" and select “Patents”)**

No, I have no relevant interests of this type

j. **Fiduciary Officer or Other Board Membership (If you need to add an interest, please scroll to the top of the page, click "add interest" and select “Fiduciary Officer”)**

No, I have no relevant interests of this type

k. **Stock or stock options (If you need to add an interest, please scroll to the top of the page, click "add interest" and select the appropriate interest type)**

No, I have no relevant interests of this type

l. **Support for attending meetings or other travel (If you need to add an interest, please scroll to the top of the page, click "add interest" and select “Travel”)**

No, I have no relevant interests of this type

4. **Was any individual paid to provide professional writing assistance with this manuscript?**

No.

5. **Have you or your institution received equipment, materials, drugs, or services in direct support of the work in the manuscript (without time limit) not disclosed above?**

No.

6. **In the past 36 months, have you received equipment, materials, drugs, medical writing, gifts or other services from for-profit or not-for-profit third parties whose interests may be affected by the content of the manuscript not disclosed above?**

No.

7. **Are there other financial or non-financial interests that readers could perceive to have influenced, or that give the appearance of potentially influencing, what you wrote in the submitted work not disclosed above.**

No.

Certification

I certify that I have answered every question and the information provided in this disclosure is complete and accurate.

Convey Global Disclosure System | AAMC

David Glynn

Discloser Identifier: 55024045
Disclosure Purpose: VIH-2025-0718.R1 - Value-based Pricing of Health Services with Health Equity Considerations.

Employment Information: Currently Employed

Summary of Interests

Company or Organization

Entity	Type	Relevant to this Disclosure
**National University of Ireland, Galway**	Employment Current Employment	

Title: Research Fellow

Additional Questions

1. **Please select which of the following apply to each relationship or activity:**
   a. **Employment** National University of Ireland, Galway

      The relationship is in direct support of the work reported in the manuscript anytime from when the work was conceived

2. **I confirm I have disclosed all direct support for the present manuscript (e.g. funding, provision of study materials, medical writing, article processing charges, etc.) There is no time limit for this item.**

   Yes

3. **Please indicate below whether in the past 36 months you have had any of the following interests that are topically related to the work reported in the manuscript.**
   a. **Employment (If you need to add an interest, please scroll to the top of the page, click "add interest" and select “Employment”)**

      Yes, as disclosed above

   b. **Grants or contracts for research (If you need to add an interest, please scroll to the top of the page, click "add interest" and select “Grant/Contract”)**

      No, I have no relevant interests of this type

   c. **Payment for consulting (If you need to add an interest, please scroll to the top of the page, click "add interest" and select “Independent Contractor”)**

      No, I have no relevant interests of this type

   d. **Payments or honoraria for lectures, presentations, speakers bureaus, or educational events (If you need to add an interest, please scroll to the top of the page, click "add interest" and select "Independent Contractor" and include the correct information under "Consultant")**

      No, I have no relevant interests of this type

   e. **Payment for service on an advisory board (If you need to add an interest, please scroll to the top of the page, click "add interest" and select “Independent Contractor,” and choose “Other”)**

      No, I have no relevant interests of this type

   f. **Payment for participation Data and safety monitoring board (If you need to add an interest, please scroll to the top of the page, click "add interest" and select “Independent Contractor”)**

      No, I have no relevant interests of this type

   g. **Expert witness testimony (If you need to add an interest, please scroll to the top of the page, click "add interest" and select “Independent Contractor”)**

      No, I have no relevant interests of this type

h. **Royalties from Patents, Trademarks, Copyrights or other intellectual property (If you need to add an interest, please scroll to the top of the page, click "add interest" and select the appropriate interest type)**

No, I have no relevant interests of this type

i. **Patents planned, issued, or pending, whether or not you receive royalties (If you need to add an interest, please scroll to the top of the page, click "add interest" and select "Patents")**

No, I have no relevant interests of this type

j. **Fiduciary Officer or Other Board Membership (If you need to add an interest, please scroll to the top of the page, click "add interest" and select "Fiduciary Officer")**

No, I have no relevant interests of this type

k. **Stock or stock options (If you need to add an interest, please scroll to the top of the page, click "add interest" and select the appropriate interest type)**

No, I have no relevant interests of this type

l. **Support for attending meetings or other travel (If you need to add an interest, please scroll to the top of the page, click "add interest" and select "Travel")**

No, I have no relevant interests of this type

4. **Was any individual paid to provide professional writing assistance with this manuscript?**

No.

5. **Have you or your institution received equipment, materials, drugs, or services in direct support of the work in the manuscript (without time limit) not disclosed above?**

No.

6. **In the past 36 months, have you received equipment, materials, drugs, medical writing, gifts or other services from for-profit or not-for-profit third parties whose interests may be affected by the content of the manuscript not disclosed above?**

No.

7. **Are there other financial or non-financial interests that readers could perceive to have influenced, or that give the appearance of potentially influencing, what you wrote in the submitted work not disclosed above.**

No.

Certification

I certify that I have answered every question and the information provided in this disclosure is complete and accurate.

Convey Global Disclosure System | AAMC

Nils Gutacker

Discloser Identifier: 55024503
Disclosure Purpose: VIH-2025-0718.R1 - Value-based Pricing of Health Services with Health Equity Considerations.

Employment Information: Currently Employed

Summary of Interests

Company or Organization

Entity	Type	Relevant to this Disclosure
**National Institute for Health Research**	Grant / Contract	Yes
**University of York**	Employment Current Employment	Yes
**Title:** Professor		

Additional Questions

1. **Please select which of the following apply to each relationship or activity:**
   a. **Employment** University of York

      The relationship is in direct support of the work reported in the manuscript anytime from when the work was conceived

   b. **Grant / Contract** National Institute for Health Research

      The relationship is in direct support of the work reported in the manuscript anytime from when the work was conceived

2. **I confirm I have disclosed all direct support for the present manuscript (e.g. funding, provision of study materials, medical writing, article processing charges, etc.) There is no time limit for this item.**

   Yes

3. **Please indicate below whether in the past 36 months you have had any of the following interests that are topically related to the work reported in the manuscript.**
   a. **Employment (If you need to add an interest, please scroll to the top of the page, click "add interest" and select “Employment”)**

      Yes, as disclosed above

   b. **Grants or contracts for research (If you need to add an interest, please scroll to the top of the page, click "add interest" and select “Grant/Contract”)**

      Yes, as disclosed above

   c. **Payment for consulting (If you need to add an interest, please scroll to the top of the page, click "add interest" and select “Independent Contractor”)**

      No, I have no relevant interests of this type

   d. **Payments or honoraria for lectures, presentations, speakers bureaus, or educational events (If you need to add an interest, please scroll to the top of the page, click "add interest" and select "Independent Contractor" and include the correct information under "Consultant")**

      No, I have no relevant interests of this type

   e. **Payment for service on an advisory board (If you need to add an interest, please scroll to the top of the page, click "add interest" and select “Independent Contractor,” and choose “Other”)**

      No, I have no relevant interests of this type

   f. **Payment for participation Data and safety monitoring board (If you need to add an interest, please scroll to the top of the page, click "add interest" and select “Independent Contractor”)**

      No, I have no relevant interests of this type

g. **Expert witness testimony (If you need to add an interest, please scroll to the top of the page, click "add interest" and select “Independent Contractor”)**

No, I have no relevant interests of this type

h. **Royalties from Patents, Trademarks, Copyrights or other intellectual property (If you need to add an interest, please scroll to the top of the page, click "add interest" and select the appropriate interest type)**

No, I have no relevant interests of this type

i. **Patents planned, issued, or pending, whether or not you receive royalties (If you need to add an interest, please scroll to the top of the page, click "add interest" and select “Patents”)**

No, I have no relevant interests of this type

j. **Fiduciary Officer or Other Board Membership (If you need to add an interest, please scroll to the top of the page, click "add interest" and select “Fiduciary Officer”)**

No, I have no relevant interests of this type

k. **Stock or stock options (If you need to add an interest, please scroll to the top of the page, click "add interest" and select the appropriate interest type)**

No, I have no relevant interests of this type

l. **Support for attending meetings or other travel (If you need to add an interest, please scroll to the top of the page, click "add interest" and select “Travel”)**

No, I have no relevant interests of this type

4. **Was any individual paid to provide professional writing assistance with this manuscript?**

No.

5. **Have you or your institution received equipment, materials, drugs, or services in direct support of the work in the manuscript (without time limit) not disclosed above?**

No.

6. **In the past 36 months, have you received equipment, materials, drugs, medical writing, gifts or other services from for-profit or not-for-profit third parties whose interests may be affected by the content of the manuscript not disclosed above?**

No.

7. **Are there other financial or non-financial interests that readers could perceive to have influenced, or that give the appearance of potentially influencing, what you wrote in the submitted work not disclosed above.**

No.

Certification

I certify that I have answered every question and the information provided in this disclosure is complete and accurate.

Convey
Global Disclosure System
AAMC

Luigi Siciliani

Oct 10, 2025 12:21 PM ET
ISPOR

Discloser Identifier: 70781991
Disclosure Purpose: VIH-2025-0718.R1 - Value-based Pricing of Health Services with Health Equity Considerations.

Employment Information: Currently Employed

Summary of Interests

Company or Organization

Entity	Type		Relevant to this Disclosure
**National Institute of Health**	Other		
**Category:** Other			
**University of York**	Employment	Current Employment	
**Title:** Professor			

Additional Questions

1. **Please select which of the following apply to each relationship or activity:**
   a. **Other Professional Activities - Other** National Institute of Health

      The relationship is in direct support of the work reported in the manuscript anytime from when the work was conceived

   b. **Employment** University of York

      The relationship is in direct support of the work reported in the manuscript anytime from when the work was conceived

2. **I confirm I have disclosed all direct support for the present manuscript (e.g. funding, provision of study materials, medical writing, article processing charges, etc.) There is no time limit for this item.**

   Yes

3. **Please indicate below whether in the past 36 months you have had any of the following interests that are topically related to the work reported in the manuscript.**
   a. **Employment (If you need to add an interest, please scroll to the top of the page, click "add interest" and select "Employment")**

      Yes, as disclosed above

   b. **Grants or contracts for research (If you need to add an interest, please scroll to the top of the page, click "add interest" and select "Grant/Contract")**

      Yes, as disclosed above

   c. **Payment for consulting (If you need to add an interest, please scroll to the top of the page, click "add interest" and select "Independent Contractor")**

      No, I have no relevant interests of this type

   d. **Payments or honoraria for lectures, presentations, speakers bureaus, or educational events (If you need to add an interest, please scroll to the top of the page, click "add interest" and select "Independent Contractor" and include the correct information under "Consultant")**

      No, I have no relevant interests of this type

   e. **Payment for service on an advisory board (If you need to add an interest, please scroll to the top of the page, click "add interest" and select "Independent Contractor," and choose "Other")**

      No, I have no relevant interests of this type

   f. **Payment for participation Data and safety monitoring board (If you need to add an interest, please scroll to the top of the page, click "add interest" and select "Independent Contractor")**

No, I have no relevant interests of this type

g. **Expert witness testimony (If you need to add an interest, please scroll to the top of the page, click "add interest" and select “Independent Contractor”)**

No, I have no relevant interests of this type

h. **Royalties from Patents, Trademarks, Copyrights or other intellectual property (If you need to add an interest, please scroll to the top of the page, click "add interest" and select the appropriate interest type)**

No, I have no relevant interests of this type

i. **Patents planned, issued, or pending, whether or not you receive royalties (If you need to add an interest, please scroll to the top of the page, click "add interest" and select “Patents”)**

No, I have no relevant interests of this type

j. **Fiduciary Officer or Other Board Membership (If you need to add an interest, please scroll to the top of the page, click "add interest" and select “Fiduciary Officer”)**

No, I have no relevant interests of this type

k. **Stock or stock options (If you need to add an interest, please scroll to the top of the page, click "add interest" and select the appropriate interest type)**

No, I have no relevant interests of this type

l. **Support for attending meetings or other travel (If you need to add an interest, please scroll to the top of the page, click "add interest" and select “Travel”)**

No, I have no relevant interests of this type

4. **Was any individual paid to provide professional writing assistance with this manuscript?**

   No.

5. **Have you or your institution received equipment, materials, drugs, or services in direct support of the work in the manuscript (without time limit) not disclosed above?**

   No.

6. **In the past 36 months, have you received equipment, materials, drugs, medical writing, gifts or other services from for-profit or not-for-profit third parties whose interests may be affected by the content of the manuscript not disclosed above?**

   No.

7. **Are there other financial or non-financial interests that readers could perceive to have influenced, or that give the appearance of potentially influencing, what you wrote in the submitted work not disclosed above.**

   No.

Certification

I certify that I have answered every question and the information provided in this disclosure is complete and accurate.

Convey Global Disclosure System | AAMC

Simon Walker

Discloser Identifier: 55035374
Disclosure Purpose: VIH-2025-0718.R1 - Value-based Pricing of Health Services with Health Equity Considerations.

Employment Information: Currently Employed

Summary of Interests

Company or Organization

Entity	Type	Relevant to this Disclosure
National Institute for Health Research	Grant / Contract	Yes
University of York	Employment Current Employment	
**Title:** Professor		

Additional Questions

1. **Please select which of the following apply to each relationship or activity:**
   a. **Employment** University of York

      The relationship is in direct support of the work reported in the manuscript anytime from when the work was conceived

   b. **Grant / Contract** National Institute for Health Research

      The relationship is in direct support of the work reported in the manuscript anytime from when the work was conceived

2. **I confirm I have disclosed all direct support for the present manuscript (e.g. funding, provision of study materials, medical writing, article processing charges, etc.) There is no time limit for this item.**

   Yes

3. **Please indicate below whether in the past 36 months you have had any of the following interests that are topically related to the work reported in the manuscript.**
   a. **Employment (If you need to add an interest, please scroll to the top of the page, click "add interest" and select "Employment")**

      Yes, as disclosed above

   b. **Grants or contracts for research (If you need to add an interest, please scroll to the top of the page, click "add interest" and select "Grant/Contract")**

      Yes, as disclosed above

   c. **Payment for consulting (If you need to add an interest, please scroll to the top of the page, click "add interest" and select "Independent Contractor")**

      No, I have no relevant interests of this type

   d. **Payments or honoraria for lectures, presentations, speakers bureaus, or educational events (If you need to add an interest, please scroll to the top of the page, click "add interest" and select "Independent Contractor" and include the correct information under "Consultant")**

      No, I have no relevant interests of this type

   e. **Payment for service on an advisory board (If you need to add an interest, please scroll to the top of the page, click "add interest" and select "Independent Contractor," and choose "Other")**

      No, I have no relevant interests of this type

   f. **Payment for participation Data and safety monitoring board (If you need to add an interest, please scroll to the top of the page, click "add interest" and select "Independent Contractor")**

      No, I have no relevant interests of this type

g. **Expert witness testimony (If you need to add an interest, please scroll to the top of the page, click "add interest" and select "Independent Contractor")**

No, I have no relevant interests of this type

h. **Royalties from Patents, Trademarks, Copyrights or other intellectual property (If you need to add an interest, please scroll to the top of the page, click "add interest" and select the appropriate interest type)**

No, I have no relevant interests of this type

i. **Patents planned, issued, or pending, whether or not you receive royalties (If you need to add an interest, please scroll to the top of the page, click "add interest" and select "Patents")**

No, I have no relevant interests of this type

j. **Fiduciary Officer or Other Board Membership (If you need to add an interest, please scroll to the top of the page, click "add interest" and select "Fiduciary Officer")**

No, I have no relevant interests of this type

k. **Stock or stock options (If you need to add an interest, please scroll to the top of the page, click "add interest" and select the appropriate interest type)**

No, I have no relevant interests of this type

l. **Support for attending meetings or other travel (If you need to add an interest, please scroll to the top of the page, click "add interest" and select "Travel")**

No, I have no relevant interests of this type

4. **Was any individual paid to provide professional writing assistance with this manuscript?**

No.

5. **Have you or your institution received equipment, materials, drugs, or services in direct support of the work in the manuscript (without time limit) not disclosed above?**

No.

6. **In the past 36 months, have you received equipment, materials, drugs, medical writing, gifts or other services from for-profit or not-for-profit third parties whose interests may be affected by the content of the manuscript not disclosed above?**

No.

7. **Are there other financial or non-financial interests that readers could perceive to have influenced, or that give the appearance of potentially influencing, what you wrote in the submitted work not disclosed above.**

No.

Certification

I certify that I have answered every question and the information provided in this disclosure is complete and accurate.

Convey
Global Disclosure System
AAMC
