# Supplementary material for: Value-Based Pricing of Health Services With Health Equity Considerations
Source: Value Health. 2026 Jun;29(6):1004–11. doi: 10.1016/j.jval.2025.12.014 (PMC13259816; doi:10.1016/j.jval.2025.12.014)
Supplement: Supplemental Material [file mmc2.docx]

**Appendix**

***Appendix A1. Equilibrium: number of patients provided with QOF process***

For a given patient with cost *k* and SES *s*, the GP has to decide whether to provide the QOF process or not. The GP payoff with no QOF process is:

$$V_{jcs}\left( i=0 \right)= \alpha H_{jcs}\left( i=0 \right)+ T_{j}-K_{j},$$

while the GP payoff with QOF process provided is:

$$V_{jcs}\left( i=1 \right)= \alpha H_{jcs}\left( i=1 \right)+ T_{j}-K_{j}+p_{jic}-k.$$

Therefore, the GP provides the QOF process if the following inequality holds:

$$V_{jcs}\left( i=1 \right)>V_{jcs}\left( i=0 \right)$$

or, more explicitly,

$$\alpha\left[ H_{jcs}\left( i=1 \right)- H_{jcs}\left( i=0 \right) \right]+p_{jic}>k.$$

This suggests that the GP will provide the QOF process if the health gain, as valued by the GP, plus the marginal revenue is above the cost of attracting the patient to the practice and providing the process.

Define $k_{jics}^{*}$ as the cost when the above equality holds with strict equality:

$$\alpha\left[ H_{jcs}\left( i=1 \right)- H_{jcs}\left( i=0 \right) \right]+p_{jic}=k_{jics}^{*} .$$

The total number of patients who receive the QOF for a given SES *s* is:

$n_{jics}^{*}=N_{jics} F_{jics}\left( k_{jics}^{*} \right)$.

Whether the number of patients with lower SES who receive the QOF process is higher or lower than those with higher SES depends on three factors: the number of eligible patients in each group, the ability to benefit and the distribution of costs. To focus ideas, suppose that the number of eligible patients is the same across deprived and rich patients. If patients who are more deprived benefit less from the QOF process, which weakens GP incentive to provide it, then it is always the case that fewer patients receive the QOF process. This is because by assumption the cost of attracting deprived patients is also higher.

The effect is instead theoretically indeterminate if more deprived patients have higher health benefit from the QOF process, which for a given distribution of costs, would lead to more deprived patients receiving it. But given that the cost of attracting patients to the GP practice is higher for more deprived patients, it can still be the case that the number of more deprived patients who receive the QOF process is smaller than for less deprived patients.

***Appendix A2. Parameterisation***

### Patient populations and QOF indicators

Eligible patients were registered with the practice on 31 March 2020. CPRD Aurum is representative of the English population in terms of age, sex and deprivation (Wolf et al., 2019) though practices are more likely to be located in urban areas (Mahadevan et al., 2022). All results were calculated for the 300 practices and scaled up to the English adult population.

### Socioeconomic deprivation

Patients were assigned to deprivation quintile groups based on the Index of Multiple Deprivation (IMD) score for their area of residence (Noble et al., 2019), which captures seven domains: income; employment; education, skills and training; health and disability; crime; housing; and living environment.

### Elasticity of GP supply

Since the QOF’s introduction, several indicators have been retired, i.e. the financial incentive was reduced to zero. Wilding et al. (2018) report achievement rates before and after indicator retirement for five QOF indicators retired in 2006, stratified by IMD quintile deprivation group. We used their results to derive elasticities of QOF achievement. These are in the range of 0.1 to 0.3 (Appendix A3, Table A1). Median elasticity was slightly more pronounced in the most-deprived than least-deprived patients (0.17 versus 0.15). We used an elasticity of 0.17 for the two most-deprived quintiles and of 0.15 for the three least-deprived quintiles.

***Appendix A3. Supply elasticities***

**Table A1. Elasticities**

| Indicator | Description | IMD1 (most deprived) | IMD2 | IMD3 | IMD4 | IMD5 (least deprived) |
| --- | --- | --- | --- | --- | --- | --- |
| CHD003 | Percentage of patients with coronary heart disease whose last measured total cholesterol (measured in the preceding 15 months) is 5mmol/l or less. | 0.18 | 0.19 | 0.17 | 0.20 | 0.16 |
| CKD002 | Percentage of patients on CKD register in whom the last blood pressure reading (in the preceding 12 months) is 140/85 mm/Hg or less. | 0.13 | 0.12 | 0.09 | 0.10 | 0.10 |
| HYP003 |  | 0.17 | 0.17 | 0.16 | 0.15 | 0.18 |
| MH004 | Percentage of patients aged 40 or over with schizophrenia, bipolar affective disorder or other psychoses who have a record of total cholesterol:hdl ratio in the preceding 12 months. | 0.29 | 0.21 | 0.23 | 0.22 | 0.27 |
| THY002 | Percentage of patients with hypothyroidism, on the register, with thyroid function tests recorded in the preceding 15 months. | 0.12 | 0.11 | 0.09 | 0.11 | 0.12 |
| Average |  | 0.18 | 0.16 | 0.15 | 0.15 | 0.16 |
| Median |  | 0.17 | 0.17 | 0.15 | 0.15 | 0.15 |

Source: Wilding et al. (2018).

***Appendix A4. Sensitivity analyses***

| **Table A2. Tornado diagrams**  *Simulation 1: DM018 +20% vs. DM014 -6.7%* | | | |
| --- | --- | --- | --- |
| Scenario | | Absolute deviation | Relative deviation |
|  | Base case | 0.000040836 | 0% |
| 1 | Supply elasticity +20% | 0.000049004 | 20% |
| 2 | Supply elasticity - 20% | 0.000032669 | -20% |
| 3 | Opportunity cost £30k per QALY | 0.000049873 | 22% |
| 4 | Opportunity costs fall equally on all IMD groups | 0.000041562 | 2% |
| 5 | Health benefit +20% | 0.000052618 | 29% |
| 6 | Health benefit -20% | 0.000029054 | -29% |
| 7 | Health benefit equal across IMD | 0.000062644 | 53% |
|  |  |  |  |
| *Simulation 2: DM018 +20% vs. COPD007 -9.8%* | |  |  |
| Scenario | | Absolute deviation | Relative deviation |
|  | Base case | 0.000038751 | 0% |
| 1 | Supply elasticity +20% | 0.000046501 | 20% |
| 2 | Supply elasticity - 20% | 0.000031001 | -20% |
| 3 | Opportunity cost £30k per QALY | 0.000046776 | 21% |
| 4 | Opportunity costs fall equally on all IMD groups | 0.000039395 | 2% |
| 5 | Health benefit +20% | 0.000049711 | 28% |
| 6 | Health benefit -20% | 0.000027790 | -28% |
| 7 | Health benefit equal across IMD | 0.000058703 | 51% |
|  |  |  |  |
| *Simulation 3: DM018 (IMD1) +20% vs. DM018 (IMD5) -28.3%* | | |  |
| Scenario | | Absolute deviation | Relative deviation |
|  | Base case | 0.000000472 | 0% |
| 1 | Supply elasticity +20% | 0.000000566 | 20% |
| 2 | Supply elasticity - 20% | 0.000000377 | -20% |
| 3 | Opportunity cost £30k per QALY | 0.000000239 | -49% |
| 4 | Opportunity costs fall equally on all IMD groups | 0.000000453 | -4% |
| 5 | Health benefit +20% | 0.000000473 | 0.3% |
| 6 | Health benefit -20% | 0.000000471 | -0.3% |
| 7 | Health benefit equal across IMD | 0.000011460 | 2329% |

| **Table A3. Simulation 1: Incentive for DM018 is increased by +20% and financed by reducing the incentive for DM014 by -6.7%** | | | | | | |
| --- | --- | --- | --- | --- | --- | --- |
|  |  |  |  |  |  |  |
| *Scenario 1: Supply elasticity +20%* | | | | | | |
|  | **Net effect of QOF change**  **(in QALYs)** | | |  | **Average health (per person) (in QALYs)** | |
| IMD group | Health effect | Expenditure effect | Change in total health (population) |  | Baseline | Change |
| Most deprived (IMD1) | 533 | -242 | 290 |  | 62.36 | 0.000034 |
| IMD2 | 662 | -201 | 461 |  | 65.52 | 0.000051 |
| IMD3 | 648 | -200 | 448 |  | 69.49 | 0.000049 |
| IMD4 | 658 | -147 | 511 |  | 70.64 | 0.000057 |
| Least deprived (IMD5) | 655 | -128 | 527 |  | 73.02 | 0.000060 |
| Total | 3156 | -918 | 2238 |  | 68.25 | 0.000050 |
|  |  |  |  |  |  |  |
| *Scenario 2: Supply elasticity -20%* | | | | | | |
|  | **Net effect of QOF change**  **(in QALYs)** | | |  | **Average health (per person) (in QALYs)** | |
| IMD group | Health effect | Expenditure effect | Change in total health (population) |  | Baseline | Change |
| Most deprived (IMD1) | 355 | -162 | 194 |  | 62.36 | 0.000023 |
| IMD2 | 441 | -134 | 308 |  | 65.52 | 0.000034 |
| IMD3 | 432 | -133 | 299 |  | 69.49 | 0.000032 |
| IMD4 | 439 | -98 | 341 |  | 70.64 | 0.000038 |
| Least deprived (IMD5) | 437 | -85 | 352 |  | 73.02 | 0.000040 |
| Total | 2104 | -612 | 1492 |  | 68.25 | 0.000034 |
|  |  |  |  |  |  |  |
| *Scenario 3: Opportunity cost £30k per QALY* | | |  |  |  |  |
|  | **Net effect of QOF change**  **(in QALYs)** | | |  | **Average health (per person) (in QALYs)** | |
| IMD group | Health effect | Expenditure effect | Change in total health (population) |  | Baseline | Change |
| Most deprived (IMD1) | 444 | -101 | 343 |  | 62.36 | 0.000041 |
| IMD2 | 552 | -84 | 468 |  | 65.52 | 0.000051 |
| IMD3 | 540 | -83 | 457 |  | 69.49 | 0.000050 |
| IMD4 | 548 | -61 | 487 |  | 70.64 | 0.000054 |
| Least deprived (IMD5) | 546 | -53 | 493 |  | 73.02 | 0.000056 |
| Total | 2630 | -382 | 2248 |  | 68.25 | 0.000051 |
|  |  |  |  |  |  |  |

| *Scenario 4: Opportunity costs fall equally on all IMD groups* | | | |  |  |  |
| --- | --- | --- | --- | --- | --- | --- |
|  | **Net effect of QOF change**  **(in QALYs)** | | |  | **Average health (per person) (in QALYs)** | |
| IMD group | Health effect | Expenditure effect | Change in total health (population) |  | Baseline | Change |
| Most deprived (IMD1) | 444 | -153 | 291 |  | 62.36 | 0.000035 |
| IMD2 | 552 | -153 | 399 |  | 65.52 | 0.000044 |
| IMD3 | 540 | -153 | 387 |  | 69.49 | 0.000042 |
| IMD4 | 548 | -153 | 395 |  | 70.64 | 0.000044 |
| Least deprived (IMD5) | 546 | -153 | 393 |  | 73.02 | 0.000045 |
| Total | 2630 | -765 | 1865 |  | 68.25 | 0.000042 |
|  |  |  |  |  |  |  |
| *Scenario 5: Health benefit from QOF care + 20%* | | | |  |  |  |
|  | **Net effect of QOF change**  **(in QALYs)** | | |  | **Average health (per person) (in QALYs)** | |
| IMD group | Health effect | Expenditure effect | Change in total health (population) |  | Baseline | Change |
| Most deprived (IMD1) | 533 | -202 | 331 |  | 62.36 | 0.000039 |
| IMD2 | 662 | -167 | 495 |  | 65.52 | 0.000054 |
| IMD3 | 648 | -167 | 481 |  | 69.49 | 0.000052 |
| IMD4 | 658 | -122 | 536 |  | 70.64 | 0.000060 |
| Least deprived (IMD5) | 655 | -106 | 549 |  | 73.02 | 0.000063 |
| Total | 3156 | -765 | 2391 |  | 68.25 | 0.000054 |
|  |  |  |  |  |  |  |
| *Scenario 6: Health benefit from QOF care -20%* | | |  |  |  |  |
|  | **Net effect of QOF change**  **(in QALYs)** | | |  | **Average health (per person) (in QALYs)** | |
| IMD group | Health effect | Expenditure effect | Change in total health (population) |  | Baseline | Change |
| Most deprived (IMD1) | 355 | -202 | 153 |  | 62.36 | 0.000018 |
| IMD2 | 441 | -167 | 274 |  | 65.52 | 0.000030 |
| IMD3 | 432 | -167 | 265 |  | 69.49 | 0.000029 |
| IMD4 | 439 | -122 | 316 |  | 70.64 | 0.000035 |
| Least deprived (IMD5) | 437 | -106 | 330 |  | 73.02 | 0.000038 |
| Total | 2104 | -765 | 1339 |  | 68.25 | 0.000030 |
|  |  |  |  |  |  |  |

| *Scenario 7: Health benefit from QOF care is that of IMD5 for all IMD groups* | | | | | |  |
| --- | --- | --- | --- | --- | --- | --- |
|  | **Net effect of QOF change**  **(in QALYs)** | | |  | **Average health (per person) (in QALYs)** | |
| IMD group | Health effect | Expenditure effect | Change in total health (population) |  | Baseline | Change |
| Most deprived (IMD1) | 805 | -202 | 603 |  | 62.36 | 0.000072 |
| IMD2 | 836 | -167 | 668 |  | 65.52 | 0.000073 |
| IMD3 | 649 | -167 | 482 |  | 69.49 | 0.000052 |
| IMD4 | 619 | -122 | 497 |  | 70.64 | 0.000055 |
| Least deprived (IMD5) | 546 | -106 | 440 |  | 73.02 | 0.000050 |
| Total | 3455 | -765 | 2690 |  | 68.25 | 0.000061 |

| **Table A4. Simulation 2: Incentive for DM018 is increased by +20% and financed by reducing the incentive for COPD007 by -9.8%** | | | | | | |
| --- | --- | --- | --- | --- | --- | --- |
|  |  |  |  |  |  |  |
| *Scenario 1: Supply elasticity +20%* | | |  |  |  |  |
|  | **Net effect of QOF change**  **(in QALYs)** | | |  | **Average health (per person) (in QALYs)** | |
| IMD group | Health effect | Expenditure effect | Change in total health (population) |  | Baseline | Change |
| Most deprived (IMD1) | 490 | -215 | 275 |  | 62.36 | 0.000033 |
| IMD2 | 614 | -178 | 436 |  | 65.52 | 0.000048 |
| IMD3 | 606 | -178 | 428 |  | 69.49 | 0.000047 |
| IMD4 | 614 | -130 | 484 |  | 70.64 | 0.000054 |
| Least deprived (IMD5) | 616 | -113 | 502 |  | 73.02 | 0.000058 |
| Total | 2940 | -815 | 2125 |  | 68.25 | 0.000048 |
|  |  |  |  |  |  |  |
| *Scenario 2: Supply elasticity -20%* | | | | | | |
|  | **Net effect of QOF change**  **(in QALYs)** | | |  | **Average health (per person) (in QALYs)** | |
| IMD group | Health effect | Expenditure effect | Change in total health (population) |  | Baseline | Change |
| Most deprived (IMD1) | 326 | -143 | 183 |  | 62.36 | 0.000022 |
| IMD2 | 409 | -119 | 290 |  | 65.52 | 0.000032 |
| IMD3 | 404 | -118 | 286 |  | 69.49 | 0.000031 |
| IMD4 | 410 | -87 | 323 |  | 70.64 | 0.000036 |
| Least deprived (IMD5) | 410 | -76 | 335 |  | 73.02 | 0.000038 |
| Total | 1960 | -543 | 1417 |  | 68.25 | 0.000032 |
|  |  |  |  |  |  |  |
| *Scenario 3: Opportunity cost £30k per QALY* | | |  |  |  |  |
|  | **Net effect of QOF change**  **(in QALYs)** | | |  | **Average health (per person) (in QALYs)** | |
| IMD group | Health effect | Expenditure effect | Change in total health (population) |  | Baseline | Change |
| Most deprived (IMD1) | 408 | -90 | 318 |  | 62.36 | 0.000038 |
| IMD2 | 512 | -74 | 437 |  | 65.52 | 0.000048 |
| IMD3 | 505 | -74 | 431 |  | 69.49 | 0.000047 |
| IMD4 | 512 | -54 | 458 |  | 70.64 | 0.000051 |
| Least deprived (IMD5) | 513 | -47 | 466 |  | 73.02 | 0.000053 |
| Total | 2450 | -340 | 2110 |  | 68.25 | 0.000047 |
|  |  |  |  |  |  |  |

| *Scenario 4: Opportunity costs fall equally on all IMD groups* | | | |  |  |  |
| --- | --- | --- | --- | --- | --- | --- |
|  | **Net effect of QOF change**  **(in QALYs)** | | |  | **Average health (per person) (in QALYs)** | |
| IMD group | Health effect | Expenditure effect | Change in total health (population) |  | Baseline | Change |
| Most deprived (IMD1) | 408 | -136 | 272 |  | 62.36 | 0.000032 |
| IMD2 | 512 | -136 | 376 |  | 65.52 | 0.000041 |
| IMD3 | 505 | -136 | 369 |  | 69.49 | 0.000040 |
| IMD4 | 512 | -136 | 376 |  | 70.64 | 0.000042 |
| Least deprived (IMD5) | 513 | -136 | 377 |  | 73.02 | 0.000043 |
| Total | 2450 | -679 | 1771 |  | 68.25 | 0.000040 |
|  |  |  |  |  |  |  |
| *Scenario 5: Health benefit from QOF care +20%* | | | | | | |
|  | **Net effect of QOF change**  **(in QALYs)** | | |  | **Average health (per person) (in QALYs)** | |
| IMD group | Health effect | Expenditure effect | Change in total health (population) |  | Baseline | Change |
| Most deprived (IMD1) | 490 | -179 | 310 |  | 62.36 | 0.000037 |
| IMD2 | 614 | -149 | 465 |  | 65.52 | 0.000051 |
| IMD3 | 606 | -148 | 458 |  | 69.49 | 0.000050 |
| IMD4 | 614 | -109 | 506 |  | 70.64 | 0.000056 |
| Least deprived (IMD5) | 616 | -94 | 521 |  | 73.02 | 0.000060 |
| Total | 2940 | -679 | 2261 |  | 68.25 | 0.000051 |
|  |  |  |  |  |  |  |
| *Scenario 6: Health benefit from QOF care -20%* | | |  |  |  |  |
|  | **Net effect of QOF change**  **(in QALYs)** | | |  | **Average health (per person) (in QALYs)** | |
| IMD group | Health effect | Expenditure effect | Change in total health (population) |  | Baseline | Change |
| Most deprived (IMD1) | 326 | -179 | 147 |  | 62.36 | 0.000017 |
| IMD2 | 409 | -149 | 261 |  | 65.52 | 0.000029 |
| IMD3 | 404 | -148 | 256 |  | 69.49 | 0.000028 |
| IMD4 | 410 | -109 | 301 |  | 70.64 | 0.000033 |
| Least deprived (IMD5) | 410 | -94 | 316 |  | 73.02 | 0.000036 |
| Total | 1960 | -679 | 1281 |  | 68.25 | 0.000029 |
|  |  |  |  |  |  |  |

| *Scenario 7: Health benefit from QOF care is that of IMD5 for all IMD groups* | | | | | |  |
| --- | --- | --- | --- | --- | --- | --- |
|  | **Net effect of QOF change**  **(in QALYs)** | | |  | **Average health (per person) (in QALYs)** | |
| IMD group | Health effect | Expenditure effect | Change in total health (population) |  | Baseline | Change |
| Most deprived (IMD1) | 735 | -179 | 556 |  | 62.36 | 0.000066 |
| IMD2 | 774 | -149 | 625 |  | 65.52 | 0.000069 |
| IMD3 | 605 | -148 | 457 |  | 69.49 | 0.000050 |
| IMD4 | 579 | -109 | 470 |  | 70.64 | 0.000052 |
| Least deprived (IMD5) | 513 | -94 | 419 |  | 73.02 | 0.000048 |
| Total | 3206 | -679 | 2527 |  | 68.25 | 0.000057 |

| **Table A5. Simulation 3: Incentive for DM018 IMD1 is increased by +20% and financed by reducing the incentive for DM018 IMD5 by 28.3%** | | | | | | |
| --- | --- | --- | --- | --- | --- | --- |
|  |  |  |  |  |  |  |
| *Scenario 1: Supply elasticity +20%* | | | | | | |
|  | **Net effect of QOF change**  **(in QALYs)** | | |  | **Average health (per person) (in QALYs)** | |
| IMD group | Health effect | Expenditure effect | Change in total health (population) |  | Baseline | Change |
| Most deprived (IMD1) | 536 | 6 | 542 |  | 62.36 | 0.000064 |
| IMD2 | 0 | 5 | 5 |  | 65.52 | 0.000001 |
| IMD3 | 0 | 5 | 5 |  | 69.49 | 0.000001 |
| IMD4 | 0 | 4 | 4 |  | 70.64 | 0.000000 |
| Least deprived (IMD5) | -931 | 3 | -928 |  | 73.02 | -0.000106 |
| Total | -395 | 24 | -371 |  | 68.25 | -0.000008 |
|  |  |  |  |  |  |  |
| *Scenario 2: Supply elasticity -20%* | | | | | | |
|  | **Net effect of QOF change**  **(in QALYs)** | | |  | **Average health (per person) (in QALYs)** | |
| IMD group | Health effect | Expenditure effect | Change in total health (population) |  | Baseline | Change |
| Most deprived (IMD1) | 357 | 4 | 362 |  | 62.36 | 0.000043 |
| IMD2 | 0 | 3 | 3 |  | 65.52 | 0.000000 |
| IMD3 | 0 | 3 | 3 |  | 69.49 | 0.000000 |
| IMD4 | 0 | 3 | 3 |  | 70.64 | 0.000000 |
| Least deprived (IMD5) | -621 | 2 | -618 |  | 73.02 | -0.000071 |
| Total | -263 | 16 | -247 |  | 68.25 | -0.000006 |
|  |  |  |  |  |  |  |
| *Scenario 3: Opportunity cost £30k per QALY* | | |  |  |  |  |
|  | **Net effect of QOF change**  **(in QALYs)** | | |  | **Average health (per person) (in QALYs)** | |
| IMD group | Health effect | Expenditure effect | Change in total health (population) |  | Baseline | Change |
| Most deprived (IMD1) | 447 | 3 | 449 |  | 62.36 | 0.000053 |
| IMD2 | 0 | 2 | 2 |  | 65.52 | 0.000000 |
| IMD3 | 0 | 2 | 2 |  | 69.49 | 0.000000 |
| IMD4 | 0 | 2 | 2 |  | 70.64 | 0.000000 |
| Least deprived (IMD5) | -776 | 1 | -774 |  | 73.02 | -0.000089 |
| Total | -329 | 10 | -319 |  | 68.25 | -0.000007 |
|  |  |  |  |  |  |  |

| *Scenario 4: Opportunity costs fall equally on all IMD groups* | | | |  |  |  |
| --- | --- | --- | --- | --- | --- | --- |
|  | **Net effect of QOF change**  **(in QALYs)** | | |  | **Average health (per person) (in QALYs)** | |
| IMD group | Health effect | Expenditure effect | Change in total health (population) |  | Baseline | Change |
| Most deprived (IMD1) | 447 | 4 | 451 |  | 62.36 | 0.000054 |
| IMD2 | 0 | 4 | 4 |  | 65.52 | 0.000000 |
| IMD3 | 0 | 4 | 4 |  | 69.49 | 0.000000 |
| IMD4 | 0 | 4 | 4 |  | 70.64 | 0.000000 |
| Least deprived (IMD5) | -776 | 4 | -772 |  | 73.02 | -0.000088 |
| Total | -329 | 20 | -309 |  | 68.25 | -0.000007 |
|  |  |  |  |  |  |  |
| *Scenario 5: Health benefit from QOF care +20%* | | | | | | |
|  | **Net effect of QOF change**  **(in QALYs)** | | |  | **Average health (per person) (in QALYs)** | |
| IMD group | Health effect | Expenditure effect | Change in total health (population) |  | Baseline | Change |
| Most deprived (IMD1) | 536 | 5 | 541 |  | 62.36 | 0.000064 |
| IMD2 | 0 | 4 | 4 |  | 65.52 | 0.000000 |
| IMD3 | 0 | 4 | 4 |  | 69.49 | 0.000000 |
| IMD4 | 0 | 3 | 3 |  | 70.64 | 0.000000 |
| Least deprived (IMD5) | -931 | 3 | -928 |  | 73.02 | -0.000106 |
| Total | -395 | 20 | -375 |  | 68.25 | -0.000008 |
|  |  |  |  |  |  |  |
| *Scenario 6: Health benefit from QOF care -20%* | | | | | | |
|  | **Net effect of QOF change**  **(in QALYs)** | | |  | **Average health (per person) (in QALYs)** | |
| IMD group | Health effect | Expenditure effect | Change in total health (population) |  | Baseline | Change |
| Most deprived (IMD1) | 357 | 5 | 363 |  | 62.36 | 0.000043 |
| IMD2 | 0 | 4 | 4 |  | 65.52 | 0.000000 |
| IMD3 | 0 | 4 | 4 |  | 69.49 | 0.000000 |
| IMD4 | 0 | 3 | 3 |  | 70.64 | 0.000000 |
| Least deprived (IMD5) | -621 | 3 | -618 |  | 73.02 | -0.000071 |
| Total | -263 | 20 | -244 |  | 68.25 | -0.000005 |
|  |  |  |  |  |  |  |

| *Scenario 7: Health benefit from QOF care is that of IMD5 for all IMD groups* | | | | | | |
| --- | --- | --- | --- | --- | --- | --- |
|  | **Net effect of QOF change**  **(in QALYs)** | | |  | **Average health (per person) (in QALYs)** | |
| IMD group | Health effect | Expenditure effect | Change in total health (population) |  | Baseline | Change |
| Most deprived (IMD1) | 810 | 5 | 815 |  | 62.36 | 0.000097 |
| IMD2 | 0 | 4 | 4 |  | 65.52 | 0.000000 |
| IMD3 | 0 | 4 | 4 |  | 69.49 | 0.000000 |
| IMD4 | 0 | 3 | 3 |  | 70.64 | 0.000000 |
| Least deprived (IMD5) | -776 | 3 | -773 |  | 73.02 | -0.000089 |
| Total | 34 | 20 | 54 |  | 68.25 | 0.000001 |
